# Retraction: The angiotensin receptor neprilysin inhibitor LCZ696 attenuates renal fibrosis via ASK1/JNK/p38 MAPK-mediated apoptosis in unilateral ureteral obstruction

**DOI:** 10.1371/journal.pone.0340045

**Published:** 2026-01-07

**Authors:** The PLOS One Editors

After this article [1] was published, concerns were raised about Figs 1–6.

Specifically:

- The following panels appear to partially or fully overlap, when aspect ratio and/or color levels are altered:
  - The UUO+VAL and UUO+GS panels in Fig 1C of [1], and Fig 3J in [2].
  - The Sham panels in Fig 1C and Fig. 2B of [1], and the Sham panel in Fig 1E of [3].
  - The UUO+LCZ panel in Fig 2B of [1] and the UUO+GSK872 panel in Fig. 3C of [4] (retracted in [5]).
  - The Sham panel in Fig 4B of [1] and Fig. 7A of [6].
  - The UUO and UUO+GS panels in Fig 6A.
  - The p-MEK4 and p-p38 panels in Fig 6D.
- When levels are adjusted to visualize the background of the western blot panels in Figs 1D, 2C, 3A, 4C, 5C, 6C and 6D, multiple regions appear to be discontinuous with adjacent background areas.

The authors did not respond to editorial queries about these concerns. In the absence of underlying data, the above issues remain unresolved.

In light of the above unresolved concerns, which question the reliability and integrity of the reported results and conclusions, the *PLOS One* Editors retract this article.

All authors either did not respond directly or could not be reached.

The UUO + VAL and UUO + GS panels in Fig 1C, the UUO + LCZ panel in Fig 2B, and the Sham panel in Fig 4B in [1] appear similar to content previously published in [2,4,6] under a CC BY 4.0 license. The UUO + VAL and UUO + GS panels in Fig 1C, the UUO + LCZ panel in Fig 2B, and the Sham panel in Fig 4B are subject to the license that applies to the original articles.
